# Gamma knife radiosurgery for essential tremor: A Case report and review of the literature

**DOI:** 10.1186/1477-7819-8-20

**Published:** 2010-03-22

**Authors:** Ameer L Elaimy, John J Demakas, Benjamin J Arthurs, Barton S Cooke, Robert K Fairbanks, Wayne T Lamoreaux, Alexander R Mackay, David R Greeley, Christopher M Lee

**Affiliations:** 1 Gamma Knife of Spokane, 910 W 5th Ave, Suite 102, Spokane, WA 99204, USA; 2 Carroll College, Department of Natural Sciences, 1601 N Benton Ave, Helena, MT 59625, USA; 3 University of Washington School of Medicine, 325 9thAve, Seattle, WA 98104, USA; 4 Spokane Brain & Spine, 801 W 5thAve, Suite 210, Spokane, WA 99204, USA; 5 Cancer Care Northwest, 910 W 5th Ave, Suite 102, Spokane, WA 99204, USA; 6 MacKay & Meyer MDs, 711 S Cowley St, Suite 210, Spokane, WA 99202, USA; 7 Northwest Neurological PLLC, 507 S Washington St, Suite 101, Spokane, WA 99204, USA

## Abstract

Approximately 5 million people in America are affected by essential tremors (ET), which are classified as a type of *benign *movement disorder. This disease manifests as tremors that usually occur in the hands, but they may also be present in the head, face, tongue, and lower limbs. Radiofrequency thalamotomy (RF) and deep brain stimulation (DBS) are common invasive procedures with proven track records that are used to treat ET. Although these procedures have high success rates, they still put patients at risk of potential side effects and are invasive by nature. Thalamotomy using the gamma knife (GK) also produces favorable outcomes in treating tremors, without the complications associated with invasive neurosurgery procedures. This report describes the presenting symptoms and extended treatment outcome for a patient with an advanced case of ET, who received GK thalamotomy treatment six years ago. Because of this non-invasive treatment, she regained the ability to paint and live with an improved quality of life. We also discuss and review the relevant literature regarding the risks and benefits of this treatment modality. GK thalamotomy is one effective option for the treatment of ET, and due to its noninvasive nature, it has a different risk profile than neurosurgery. We suggest that GK thalamotomy should be presented as one viable treatment option to all ET patients, and should be recommended to those who would be best served by less invasive treatment techniques.

## Background

Essential tremor is a common type of movement disorder that normally affects people over the age of 65; however, this illness can occur in younger patients as well. In recent years, ET has been categorized as a heritable condition, which can be transferred to family members in an autosomal dominant fashion [1]. The primary symptom of ET involves shaking of the hands during voluntary movements, but it may also present with movements of the head, face, tongue, and lower limbs [1-3]. Other than tremors, there are no other direct medical symptoms associated with ET and it does not decrease life expectancy. However, many patients with ET have difficulties accomplishing their daily tasks or other activities that affect quality of life, which is how this disorder elicits a negative impact on the social and mental wellness of the patients who bear this illness [1,4].

There are multiple treatment options for ET patients. The most common medications utilized are beta-blockers. Unfortunately, these are contraindicated for many patients with asthma, diabetes, and certain heart conditions. Anti-seizure medications and botulinum toxin injections are also used, but they are known to cause unwanted side effects. Stereotactic RF thalamotomy is the most common neurosurgical procedure for treating ET. It involves MR imaging of the thalamic target (ventralis intermedius nucleus), placement of an electrode neurosurgically, stimulation of the target, and creation of a lesion through tissue ablation [5]. DBS is also an invasive surgical procedure performed as an alternative to RF thalamotomy. DBS involves the implantation of a device that utilizes electrical impulses to block abnormal nerve signals [5,6].

Even though surgical treatments such as RF thalamotomy and DBS are effective in many patients with ET, there are those who are not qualified candidates for invasive neurosurgery because of comorbid medical conditions. These include patients who use anticoagulants, who have advanced cardiac or respiratory disease, who are known to be noncompliant, and who are of advanced age. An alternative for such patients is thalamotomy using GK radiosurgery. GK thalamotomy is a safe alternative to invasive neurosurgery, and evidence shows it is successful in the treatment of ET and similar movement disorders [5,7-11]. Also, because this disorder often occurs at a late age, and pharmaceuticals can have significant side effects, GK can be the only treatment option for this population of medication-intolerable patients.

We present an inspiring case of an ET patient, whose daily life was drastically modified by the severity of her hand tremors, until GK thalamotomy treatment restored her ability to control movement and pursue her passion of painting.

## Case Presentation

### Case Report

**Figure 1:**
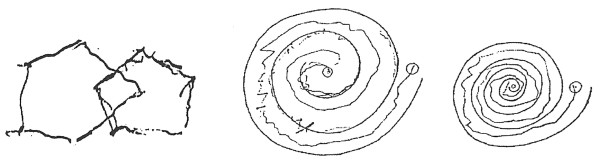
**Handwriting sample before radiosurgery**.

The patient was a 65 year-old right-handed female, who reported a history of right-handed tremors for approximately one to two years before her GK consultation in 2003. The tremors involving her right arm were fine postural tremors, as well as intentional tremors. She did not experience resting tremors. Also, the patient experienced fine tremors in her left arm. She found that the tremors were more pronounced with stress. Eventually, the patient's handwriting worsened to the point where she was no longer able to write legibly (See Fig. 1). The patient worked as a nurse, and said certain aspects of her job became difficult to accomplish (e.g. administering IVs) because of her tremor. The patient was initially treated medically for her tremor, but she did not experience significant relief and thus sought out other treatment options.

**Figure 2:**
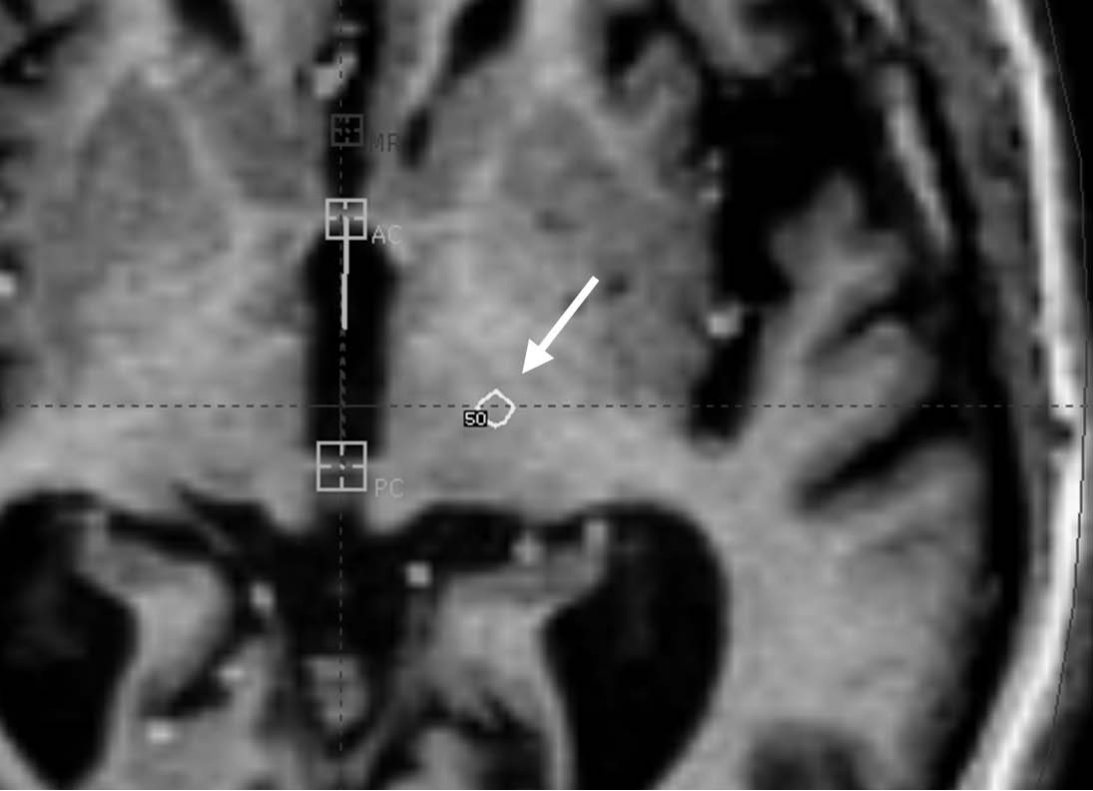
**Treatment fields; arrow specifies field**.

She consulted with a neurosurgeon, with the goal of learning the risks and benefits of the available surgical procedures. In her case, DBS was felt to be a better option than RF thalamotomy. She was educated about the risks and benefits of DBS and GK thalamotomy and opted to proceed with GK treatment. After an MRI was obtained and GK planning was complete, the patient underwent a left VIM thalamotomy, with a prescribed maximum dose of 140 Gy. The dose administered was 70 Gy to the 50% isodose line, with a 4 mm gamma knife shot. Following the procedure, the patient was monitored closely and had serial follow-up appointments with a neurosurgeon. We have included an illustration of her treatment fields (See Fig. 2).

**Figure 3:**
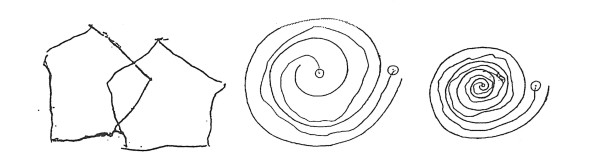
**Handwriting sample after radiosurgery**.

**Figure 4:**
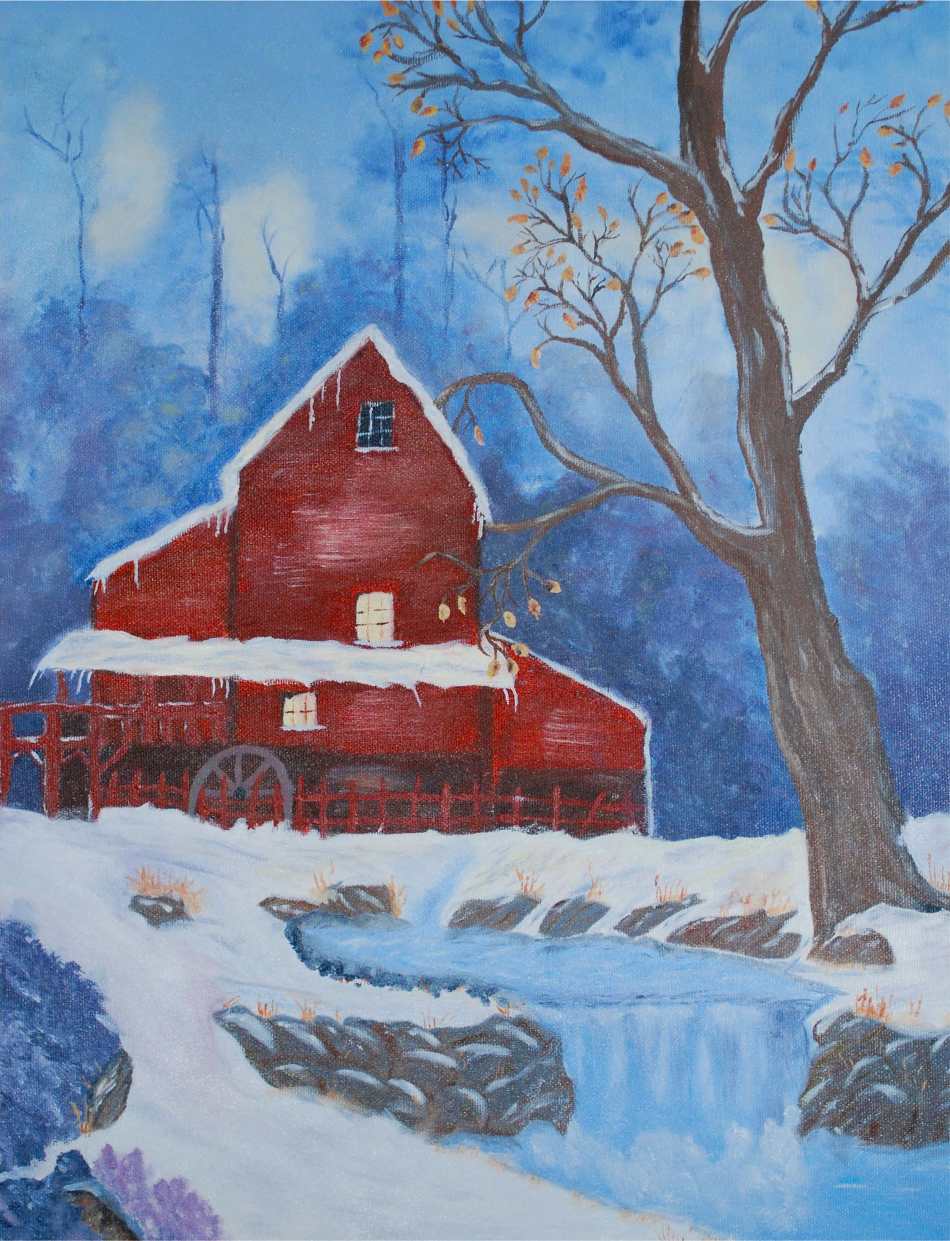
**Painting patient gave to her treating neurosurgeon**.

**Figure 5:**
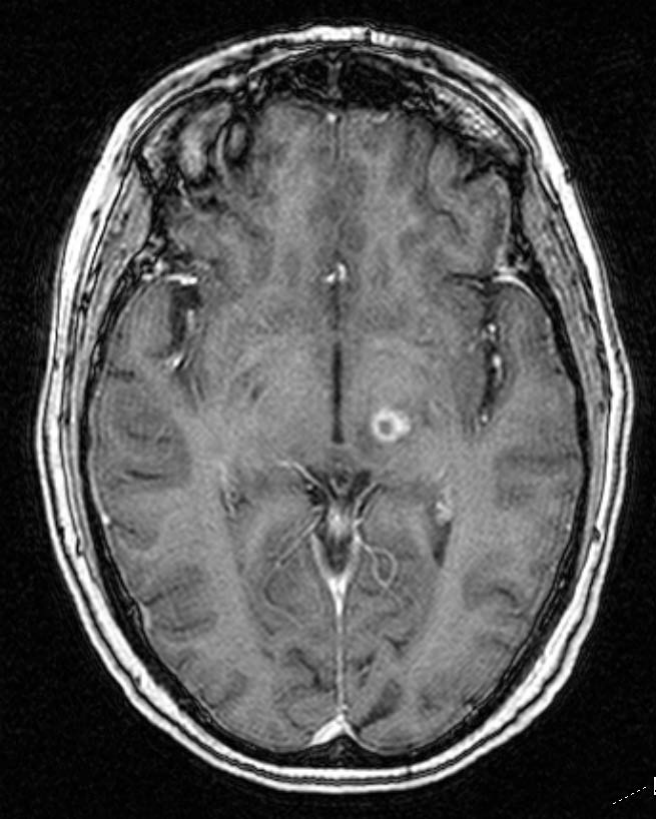
**MRI 8 months after radiosurgery**.

The patient did well post-operatively and experienced no side effects or focal neurological problems. The patient first observed tremor improvement within two weeks of radiosurgery. Tremor control continued to improve over the next eight months. At that point, the patient's tremors completely dissipated (See Fig. 3). Because of her profound improvement, the patient painted a beautiful picture for her treating neurosurgeon out of gratitude for her regained ability (See Fig. 4). United Parkinson's Disease Rating Scale (UPDRS) scoring and the Fahn-Tolosa clinical rating scale were utilized to provide an objective measurement of response to treatment [12,13]. We compared scores from pre-treatment with scores eight months after treatment to demonstrate her clinical improvements. Assessment by the treating neurosurgeon revealed that the patient's scoring improved from a grade of 3 to 0, with respect to handwriting and tremor control in both the UPDRS and the Fahn-Tolosa scales. It was also concluded that the patient's drawing capability improved from a grade of 4 to 0 by Fahn-Tolosa grading. A post-treatment MRI eight month after radiosurgery showed an 11 mm enhancing ring-like lesion consistent with the treatment (See Fig. 5).

**Figure 6:**
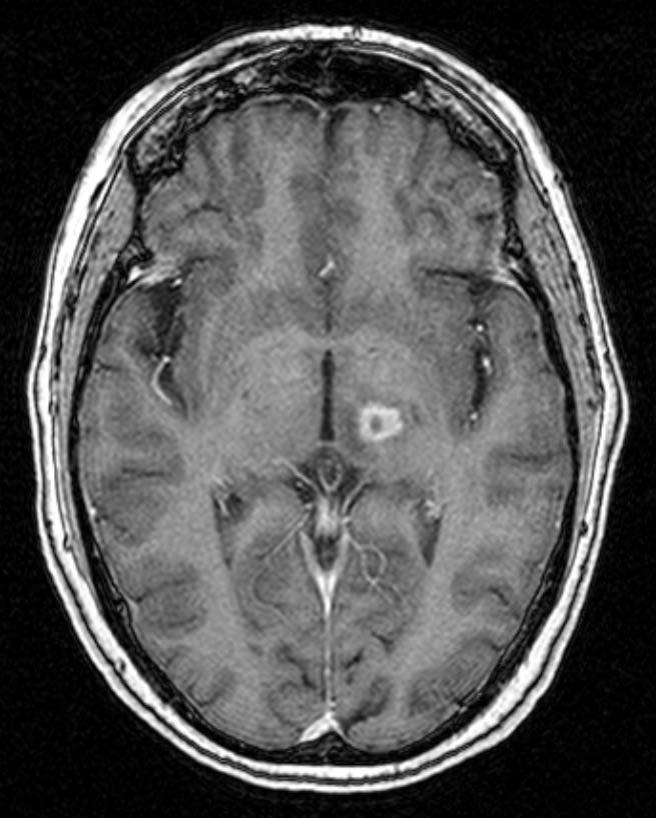
**MRI 1 year after radiosurgery**.

Unfortunately, the patient did experience complications one year after GK surgery. She developed numbness in the first three fingers of her right hand and her tongue, which led to dysarthria. The patient also reported balance problems at this time. An MRI showed increased signal in the thalamus, but no new lesions were found. The lesion measured about 10 mm in circumference, with a vertical diameter of 7 mm (See Fig. 6). A Medrol Dosepak was prescribed to the patient for the inflammation. She saw improvements in her speech and balance when she began the medication, but those issues returned shortly after completion of the dose pack. She was then placed on a Decadron taper for seven weeks, and responded quite well following that course of treatment. She saw a definite improvement in her speech and balance; however, she occasionally experienced transient numbness in her lips and the first two fingers of her right hand. The patient's status continued to improve from there on. At 72 months post treatment, her tremors have not returned and she is living a happy and fulfilling life. She still enjoys painting as a hobby.

### Review of Relevant Literature

We have reached a point in the field of neurosurgery where minimally-invasive procedures exhibit outcomes comparable to open-skull surgery. Young, et al. [11] performed a study where 27 patients with a variety of tremor causes (Parkinson's disease, ET, cerebral infarctions, and encephalitis) underwent GK thalamotomy for tremor treatment. Out of these 27 patients, 24 (88.9%) saw positive results. Specifically, 19 of these patients experienced complete tremor resolution and the other 5 had nearly complete tremor resolution, with a mean follow-up of 22.2 months. There were no complications observed. Friedman, et al. [8] conducted 15 thalamotomies using the GK. Out of these 15 patients, 14 (93.3%) experienced complete absence or a slight residual tremor three months after surgery. Similar to our patient in this case presentation, it was reported that two patients in this study exhibited severe edema three months following radiosurgery. Both of these patients underwent steroid therapy, and it was found that one of these patients significantly improved, while the second patient experienced moderate improvement, with a slight residual deficit. Plowman [14] classified post- radiation reactions into categories based on the timeframe following radiosurgery in which they occur. This is an example of a subacute tissue reaction, due to the fact that the edema occurred 3-10 months after radiosurgery. Subacute reactions are many times either completely or partially reversible. Acute reactions, occurring 12-48 hours after surgery, are rarely seen in thalamotomies with the GK because the effects of radiosurgery take time to manifest clinical symptoms.

GK thalamotomy is also an accepted treatment for tremors caused by PD. Duma, et al. [7] created 42 radiosurgical thalamic lesions in 38 PD patients with GK thalamotomy and obtained promising results. The tremor was eliminated completely in 10 thalamotomies (24%). Excellent improvement was seen in 11 (26%), good improvement was seen in 13 (31%), and mild improvement was seen in 4 (9.5%). GK treatment did not affect 4 (9.5%) patient's tremors. Therefore, 38 out of the 42 (90%) thalamotomies were deemed successful. However, one patient reportedly suffered a mild acute dysarthria one week after treatment.

Both RF thalamotomy and DBS have excellent reported control rates, but a varied side effect profile. Fox, et al. [15] reported a 91% success rate and Jankovic, et al. [16] reported a 90% success rate with open RF thalamotomy. Despite the high success rates, RF techniques put the patient at risk for intracerebral or extracerebral hemorrhage, seizures, infection, brain displacement, tension pnemocephalus, and direct injury from probe placement [11]. Unemura, et al. [6] performed a thorough review that evaluated the morbidity and mortality related to DBS. They noted 16 serious adverse events related to surgery in 14 (12.8%) of 109 patients. These included pulmonary embolism, subcortical hemorrhage, chronic SDH, venous infarction, seizure, infection, cerebrospinal fluid leak, skin erosion, and death.

A study done to compare GK thalamotomy, RF thalamotomy, and DBS by Niranjan, et al. [5] displayed confidence in all three methods, with respect to tremor control rates. DBS systems were implanted in 11 patients, who all had excellent control of their tremors immediately after surgery. Long-term follow up showed that 9 out of 11 patients maintained excellent tremor control. Out of the 15 patients that underwent GK thalamotomy, the 12 who had more than six months of follow-up showed positive results. There were no immediate complications after radiosurgery. Although, one patient who had experienced a reduction in her ET 2 months after GK treatment noted mild weakness in the contralateral arm and leg, along with dysarthria 8 months after surgery. Fortunately, the patient was managed on corticosteroids and showed incremental improvements over time, with regard to those clinical symptoms. Immediately after surgery, all 13 RF thalamotomy patients experienced improvements, but 6 of those patient's tremors reoccurred. Young, et al. [10] monitored GK thalamotomy patients for a longer period of time. At one year, 92.1% of their ET patients were completely or nearly tremor free. At four years, 88.2% maintained their radiosurgical success.

Even though invasive surgery approaches show immediate results, the amount of risk associated with treatment appears to be greater than radiosurgery using the GK. However, GK thalamotomy has its own challenges. Ohye, et al. [17] reported that in most of their 31 GK thalamotomy patients, the reduction of tremor started approximately one year after irradiation. That time period may not be preferred by some patients. Another challenge is variability of the thalamic reaction and subsequent side effects between patients. They concluded that there are two types of thalamic lesions formed by GK radiation. The first type is simple, showing a round punched-out lesion, consisting of an oval, low signal area surrounded by a ring-like high signal area. The second type is an irregular-shaped high signal zone that is large and amorphous. There was no correlation between the two types of lesions and the clinical effect on tremor. The type and volume of a thalamic lesion cannot be predicted before radiosurgery. In some cases, the lesion may extend into the internal capsule or medial thalamic region, which is usually accompanied by streaking, thus, can cause severe delayed-onset complications.

## Conclusions

We described a patient successfully treated for ET with the GK. Six years after treatment, this patient is still exceptionally pleased with her results. She has recently reported that her tremors are completely controlled; allowing her to live an active lifestyle. This patient, who previously could not write her own name legibly, is now an avid painter. The published data, as reviewed in this manuscript, indicates that GK thalamotomy is an effective procedure in the treatment of tremors and that the risk profile is different and in many cases preferable to open-skull surgery. We suggest that GK thalamotomy should be presented as an acceptable treatment option to all ET patients before making a decision to undergo invasive neurosurgery. We look forward to continued research and evolution of this exciting treatment option for patients suffering from tremors.

## List of Abbreviations

DBS: deep brain stimulation; ET: essential tremor; GK: gamma knife; PD: Parkinson's disease; RF: radiofrequency; UPDRS: United Parkinson's Disease Rating Scale; VIM: ventralis intermedius nucleus.

## Consent

Written informed consent was obtained from the patient for publication of this case report and any accompanying images.

## Competing interests

The authors declare that they have no competing interests.

## Authors' contributions

ALE and CML reviewed relevant clinical data for this case report, reviewed the current literature, and drafted the manuscript. RKF, JJD, ARM, WTL, BSC, BJA, and DRG provided clinical expertise and participated in drafting the manuscript. DRG and JJD provided clinical expertise relevant to the case report. All authors read and approved the final manuscript.
